# Radical Cystectomy for Intradiverticular Bladder Carcinoma: A Case Report

**DOI:** 10.31729/jnma.6228

**Published:** 2021-10-31

**Authors:** Anil Kumar Sah, Bipin Maharjan, Mahesh Bahadur Adhikari, Reena Rana, Sunila Basnet, Rajesh Panth, Gopi Aryal

**Affiliations:** 1 Department of Urology, Nepal Mediciti Hospital, Bhainsepati, Lalitpur, Nepal; 2 Department of Pathology, Nepal Mediciti Hospital, Bhainsepati, Lalitpur, Nepal

**Keywords:** *bladder*, *cystectomy*, *diverticulum*, *radical*, *urothelial carcinoma*

## Abstract

Herniation of bladder mucosa through the bladder wall muscle layer is known as bladder diverticulum. The incidence of bladder diverticulum is 1.7. About 0.8 to 10% of the urinary bladder diverticulum develops carcinoma. Transitional cell carcinoma is the most common. Painless hematuria is the most common clinical presentation. Different imaging modalities along with cystoscopy are the key to accurate diagnosis and staging. High grade multifocal urothelial carcinoma in the bladder diverticulum is better managed by radical cystectomy and standard pelvic lymph node dissection with an ileal conduit. Here we report a case of a 66-year old gentleman of high grade multifocal urothelial carcinoma in bladder diverticulum managed with radical cystectomy and standard pelvic lymph node dissection with an ileal conduit. Such cases have been addressed adequately in the literature, but we did not find such cases from our country.

## INTRODUCTION

Out pouching of the bladder mucosa through the weak part of the urinary bladder muscular layer is known as bladder diverticulum.^1,2^ Its incidence is not rare at all.^3^ Chronic irritation and inflammation secondary to urinary stasis in the diverticulum are responsible for neoplastic changes. Radical cystectomy is indicated for high-grade tumours and is a safe and effective procedure in the treatment of diverticular tumours.^1^ We reported a case of a 66-year gentleman who presented with painless hematuria and was later diagnosed with high-grade multifocal bladder diverticulum urothelial carcinoma which was successfully managed with radical cystectomy and standard pelvic lymph node dissection with an ileal conduit.

## CASE REPORT

**Figure 1:**
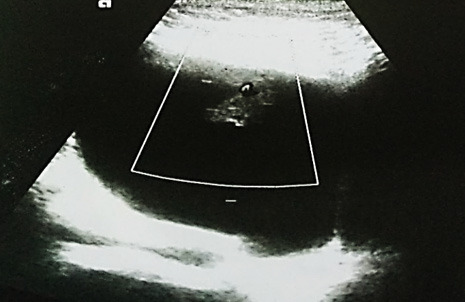
Pre-operative USG showing a polypoidal hypoechoic mass from the bladder diverticulum.

A 66-years male presented with painless occasional gross hematuria for 6months with the poor flow of urine, intermittency, abdominal straining and incomplete voiding. He was a known case of hypertension and enlarged prostate under medications. The bladder was palpable. Prostate was enlarged, firm, non-tender and non-nodular on digital rectal examination. His haemoglobin level was 12.1gm/dl with a normal kidney function test. Urine analysis showed plenty of red blood cells, pus cells 1-2/hpf with no bacterial growth on culture. Abdominal sonography showed a polypoidal hypoechoic lesion measuring 2.17x1.2cm with vascular pedicle was seen arising from the anterior nondependent portion of bladder diverticulum measuring 10.5x8.9cm (Figure 1).

The prostate is enlarged in size and measures 5.7x5.1x3.5cm which corresponds to approximately 56.1 grams in weight. Foley catheterization failed. On cystourethroscopy 1.5x1.5cm impacted urethral calculus was seen in the prostatic fossa. Pushback of the calculus in the urinary bladder failed. Therefore, pneumatic lithotripsy was done in the same region. There were multiple trabeculations, sacculations and a large diverticulum in the UB wall in the right lateral wall with multiple sessile growths and a 1.5x1.5cm size pedunculated growth inside the diverticulum.

**Figure 2:**
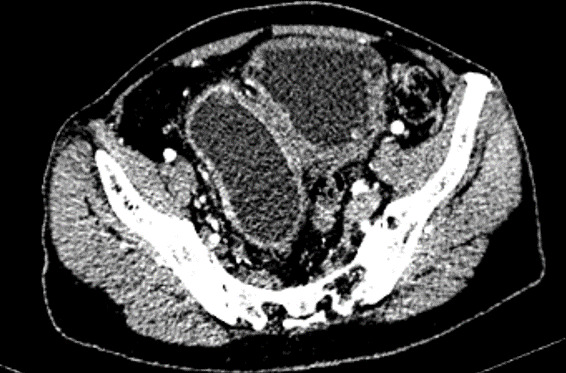
CT Scan showing a polypoidal enhancing mass arising from the bladder diverticulum.

Multiple biopsies were taken from the growths and random cold cup biopsy was taken from the proper urinary bladder wall. Histopathology came as highgrade infiltrating (lamina propria) urothelial carcinoma from the bladder diverticulum and normal findings from the bladder wall. CT abdomen showed 10.7mm wall thickness of urinary bladder with a large diverticulum measuring 9.69x4.55cm arising from the right lateral aspect of UB. There was enhancing polypoidal lesions of 13.2x11.4mm arising from the anterosuperior aspect of this diverticulum (Figure 2).

**Figure 3:**
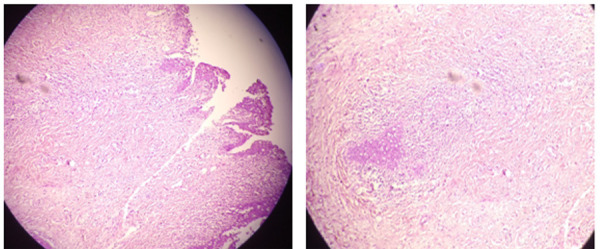
Histopathology showing urothelial carcinoma infiltrating the lamina propria with no evidence of detrusor muscle in the specimen.

Options of bladder preservative surgery and radical cystectomy with urinary diversion were discussed with the patients and the family. Finally, radical cystectomy and standard pelvic lymph node dissection (PLND) with ileal conduit were done. It was urothelial carcinoma infiltrating the lamina propria. There was no evidence of detrusor muscle in the specimen (Figure 3). The patient progressed well post-operatively and he is doing well currently on follow up.

## DISCUSSION

Urinary bladder diverticulum (BD) is an out-pouching of the bladder mucosa through the weak bladder muscle (detrusor muscle) either congenital or acquired, which may be complicated with inflammation, calculus, infection, and malignancy.^1^ The incidence of bladder diverticulum is approximately 1.7% in children and 6% in adults.^2^

Acquired diverticulum occurs as a result of raised intravesical pressure due to bladder outlet obstruction through a series of changes like trabeculations and sacculations.^3^ Congenital diverticulum is usually single that occurs as a result of the disarray of the detrusor fibres within the musculature of the bladder wall and is associated with vesicoureteric reflux and hydronephrosis. BD usually occurs in close proximity to the ureteric orifices.^4^

Chronic irritation and inflammation, secondary to urinary stasis due to lack of contractility of the diverticulum are responsible for dysplasia, leukoplakia, and squamous metaplasia. These changes are seen in almost 80% of BD.^5^

The incidence of BD tumour is 0.8 to 10%, usually occurs in the aged patients with bladder outlet obstruction, rarely on congenital diverticula. Transitional Cell Carcinoma (TCC) is the most common histological variety constituting 70-80%, followed by Squamous Cell Carcinoma (SCC) which is about 20-25% of all BD tumours. TCC along with SCC is reported in 2% of all tumours while adenocarcinoma constitutes the other 2% of these BD tumours.^6^ Painless hematuria, which accounts for about 90% is the most common clinical presentation of BD tumours, as in ordinary bladder tumours.^7^

Ultrasonography is particularly helpful in patients with contraindicated cystoscopy or unsuccessful radiographic contrast examinations due to small diverticulum ostium or occlusive tumours. However, diverticula located along with the dome or in the neck of the bladder may be more difficult to identify sonographically. BD tumours are moderately echogenic, non-shadowing mass along the wall of the diverticulum on the sonogram.

Diagnosis and staging rely on CT imaging. However, its role is limited by its inability to resolve the different layers of the bladder wall. MRI provides a better gross assessment of tumour depth especially when done with Gadolinium.^8^

Since the wall of the BD is thin, there is a high probability of penetration of the wall by diverticular tumours.^9^ Staging of the BD tumour is difficult because of the lack of muscle layer in the diverticulum. Indeed, some authors suggest skipping the T2 stage altogether when staging diverticular tumours whereas some authors suggest T1 as muscle-invasive.

Transurethral resection of the tumour is indicated in low grade, low volume Ta, Tis or T1 tumours with wide diverticular necks. It is technically challenging. It is difficult to access the diverticulum owing to a narrow neck or an acute angle of entry. There is a potential risk of bladder perforation & tumour dissemination.^9^

Diverticulectomy or Partial Cystectomy is indicated in low grade, large volume tumours with narrow diverticular necks and high-grade unifocal tumours. Partial cystectomy with PLND, followed by adjuvant intravesical immunotherapy or systemic chemotherapy, is advisable in patients with high-grade T1 tumours.^9^

Radical Cystectomy is indicated for locally advanced tumours, high-grade tumours, multifocal exophytic tumours, extensive CIS and multifocal disease with poor bladder function. It is a safe & effective procedure in the treatment of diverticular tumours.

Series of studies suggest that there is a high rate of recurrence and poor prognosis for diverticular tumours. But the newer study suggests complete tumour resection with partial cystectomy results in 5-year disease-specific survival rates of around 70%.' Complete removal is feasible for the tumours confined to the bladder diverticulum and close surveillance ensues.^1^

The ways of presentation and the modalities of the diagnosis of bladder diverticular tumours are similar to those of regular bladder tumours. Radical cystectomy with standard pelvic lymph node dissection is effectively justified for the management of high grade multifocal urothelial carcinoma of the bladder diverticulum.
